# Annotated expression and activity data for murine recombinase alleles and transgenes: the CrePortal resource

**DOI:** 10.1007/s00335-021-09909-w

**Published:** 2021-09-04

**Authors:** Michelle N. Perry, Constance M. Smith, Hiroaki Onda, Martin Ringwald, Stephen A. Murray, Cynthia L. Smith

**Affiliations:** The Jackson Laboratory, Bar Harbor, ME 04609 USA

## Abstract

Recombinase alleles and transgenes can be used to facilitate spatio-temporal specificity of gene disruption or transgene expression. However, the versatility of this in vivo recombination system relies on having detailed and accurate characterization of recombinase expression and activity to enable selection of the appropriate allele or transgene. The CrePortal (http://www.informatics.jax.org/home/recombinase) leverages the informatics infrastructure of Mouse Genome Informatics to integrate data from the scientific literature, direct data submissions from the scientific community at-large, and from major projects developing new recombinase lines and characterizing recombinase expression and specificity patterns. Searching the CrePortal by recombinase activity or specific recombinase gene driver provides users with a recombinase alleles and transgenes activity tissue summary and matrix comparison of gene expression and recombinase activity with links to generation details, a recombinase activity grid, and associated phenotype annotations. Future improvements will add cell type-based activity annotations. The CrePortal provides a comprehensive presentation of recombinase allele and transgene data to assist researchers in selection of the recombinase allele or transgene based on where and when recombination is desired.

## Introduction

**Fig. 1:**
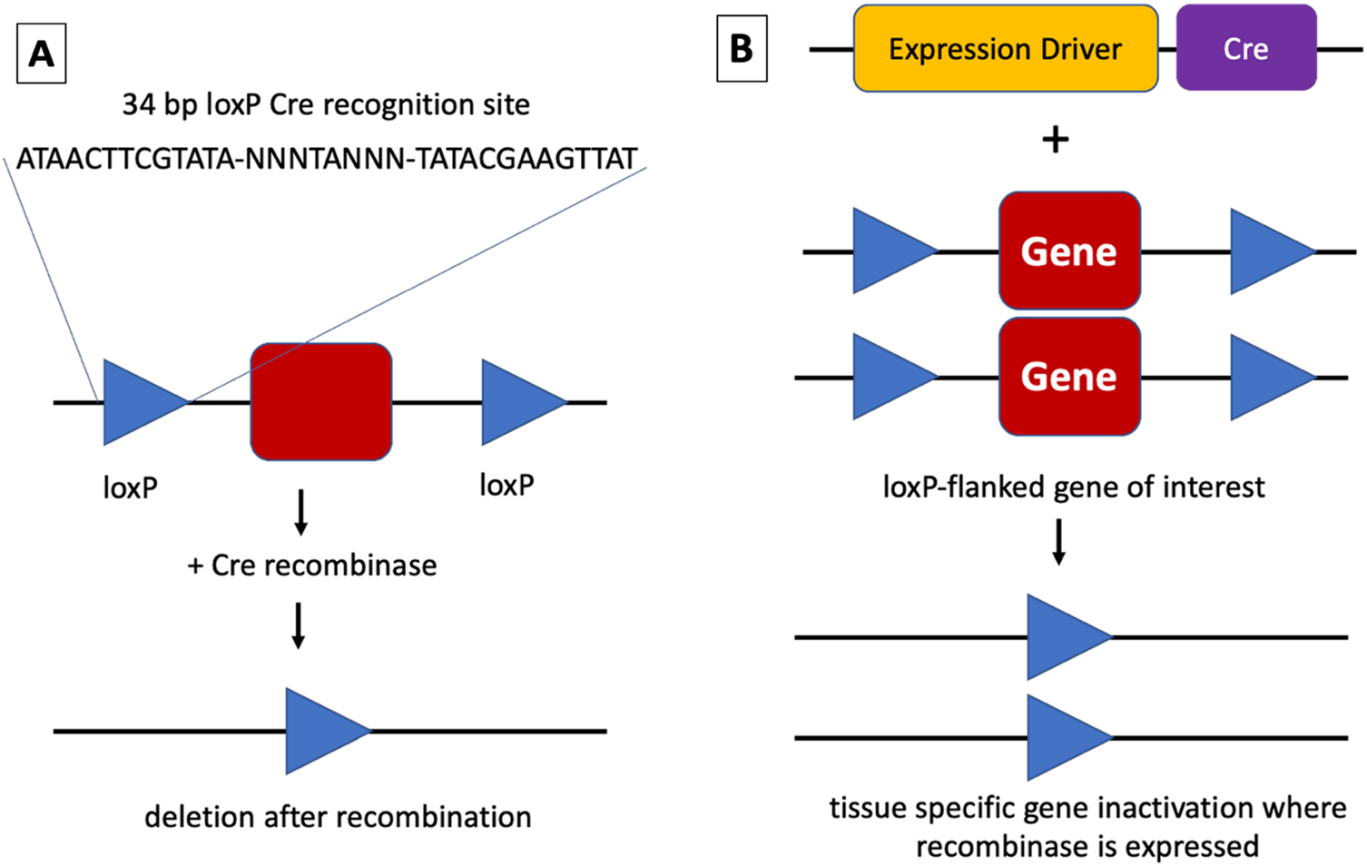
Cre recombinase in genetics. **A** Cre recombinase recognizes two repeated 34 bp loxP sites in DNA, then excises loxP-flanked sequences, leaving a single loxP site. More complex rearrangements can be made with changes to the orientation and position of the recognition sites. **B** To excise sequences in vivo, a mouse expressing Cre recombinase under the control of a tissue or temporal-specific driver is crossed with a mouse carrying LoxP sites that flank a gene or genes of interest. Progeny carrying both the loxP-flanked gene and the Cre recombinase will show deletion in the tissue of interest at the stage when the driver is expressed

Along with individual research laboratories, large-scale mouse gene targeting projects, such as KOMP, EUCOMM, NorCOMM, and TIGM (collectively, the IKMC), have delivered a vast number of conditional-ready loxP-flanked alleles to the scientific community (Skarnes et al. 2011; Bradley et al. 2012). Many of these conditional-ready alleles from large-scale projects are now available thanks to the efforts of programs such as the International Mouse Phenotyping Consortium (IMPC) and the NIH-funded KOMP2 to turn these stem cell resources into live mice (Ryder et al. 2014; Dickinson et al. 2016; Birling et al. 2021). When combined with a Cre-expressing allele, this system allows investigators to interrogate gene function through precise deletion in a temporally and tissue-specific manner (Fig. 1) (Kim et al. 2018). The value of this resource depends on the ready availability of a large, diverse set of well-characterized Cre driver lines and access to functional information about their activity.

As of June 2021, Mouse Genome Informatics (MGI; www.informatics.jax.org) has data pertaining to 3299 recombinase alleles and transgenes driven by 1230 unique promoters assayed in over 3718 tissues. Of the 3299 recombinase alleles and transgenes, 1166 are inducible. Beyond the basic cre/lox system, temporally and spatially restricted recombination can be achieved using different recombinases (e.g., D6 site-specific recombinase, dre; flp). Refinement of recombinase expression and activity can be achieved using recombinases fused with inducible elements (e.g., modified versions of the human estrogen receptor, ESR*, ERT, or ERT2) (Feil et al. 2009; Kellendonk et al. 1996; Ludwig et al. 1996; Sauer et al. 2004) or a tetracycline response element combined with a transgene expressing the reverse tet-transactivator (rtTA2; Dow et al. 2014). With the variety of tools, researchers can achieve precise recombination to refine understanding alterations in gene expression.

Characterization data that describe the timing and distribution of recombination activity in mouse lines are often fragmented in multiple publications, making it challenging for users to find critical information for good experimental design or to compare the relative utility of different driver strains for a given application. The catalog of these available mouse strains has grown in recent years. However, there are still significant gaps that limit our ability to dissect gene function in certain tissue types and developmental stages. Moreover, despite continued advances and improvements in approaches to engineer Cre driver lines, the fidelity of recombinase activity is not always ideal. Limitations reported in the function of various recombinase lines include expression in non-target tissues (“off-target” activity), inconsistent activity, incomplete or mosaic deletion in a target tissue/cell type, and/or insertional mutagenesis (Heffner et al. 2012; Perry et al. 2020). The CrePortal (http://www.informatics.jax.org/home/recombinase) is a freely accessible public database that houses comprehensive information about existing recombinase driver strains and their functional activity. This includes integrated data curated from compiled literature about these lines and via direct submissions of characterization data from resource development programs, providing the community with a comprehensive source of recombinase driver tool strains and information about them.

## Accessing data at the CrePortal web interface

**Fig. 2:**
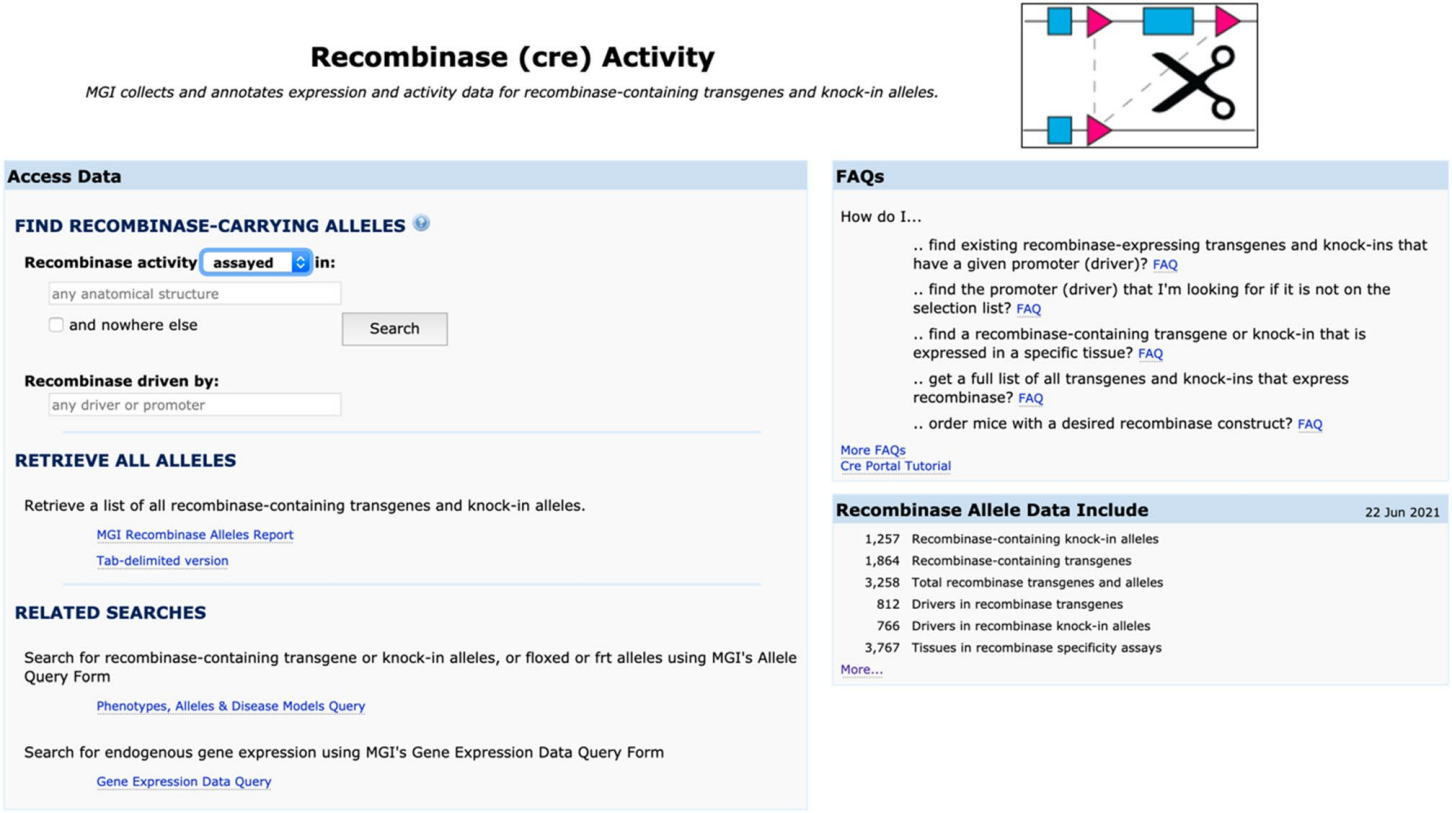
The CrePortal homepage supports searches using an anatomical structure or driver of expression or a combination of these parameters. Downloadable reports listing all recombinase alleles, the systems where activity is detected or not, and links to availability of the mouse strain are shown below the search boxes. Tutorials to assist in answering specific research questions are listed on the right. Links to help documents and other Cre resources are listed at the bottom of the page (not shown)

The CrePortal homepage (Fig. 2) offers users access to recombinase allele and transgene data via a search form and reports. In addition, the homepage provides links to related searches of phenotypes, alleles and disease models or gene expression data. Frequently asked questions point users to resources and strategies to identify their desired recombinase allele or transgene, including a Cre Portal tutorial. To identify annotated recombinase alleles and transgenes, users can search the CrePortal based on two parameters: anatomical structure and/or recombinase driver.

**Fig. 3:**
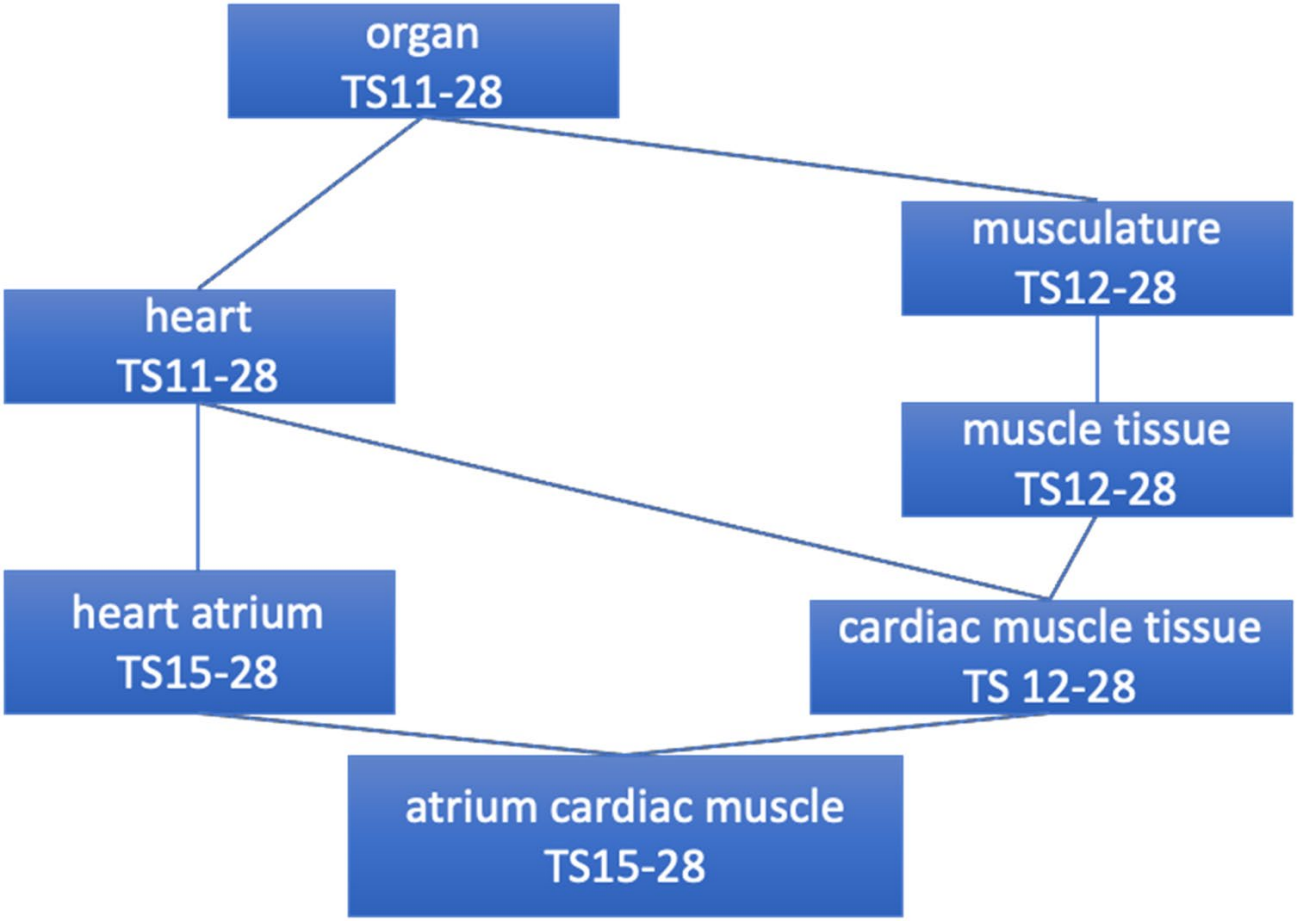
The EMAPA ontology represents the mouse anatomy for all developmental stages (including postnatal stage TS 28) and specifies which anatomical structures exist at what stages. The ontology is structured as a directed acyclic graph (DAG) so that the anatomy can be represented from different hierarchical perspectives, such as by organ system or by body region. This allows for searching and data integration at different levels of resolution. For more details on the ontology and its structure, see Hayamizu et al. (2015)

Cre reporter data represent allele patterns created through the tissue-specific activity of the Cre recombinase rather than gene expression patterns. However, from a strictly technical point of view, Cre reporter data are similar to knock-in reporter in situ data, a data type that GXD has long captured (Smith et al. 2019; Baldarelli et al. 2021). Therefore, the CrePortal takes advantage of GXD infrastructure to record and search for recombinase activity patterns. Notably, it uses the mouse developmental anatomy (EMAPA) ontology developed by the Edinburgh Mouse Atlas project (EMAP) and GXD (Hayamizu et al. 2015) to annotate the anatomical location and developmental stage of Cre activity, thus enabling hierarchical anatomical searches and the comparison of Cre activity patterns (from the CrePortal) and expression patterns (from GXD) via interactive matrix views (described further below). The EMAPA ontology is structured as a directed acyclic graph (DAG). The “abstract” representation of the anatomy is stage independent; each term within this representation has attributes describing the stage range in which that particular structure is present (Fig. 3). If a term (term A) is a direct part of another term (term B) at any time, there is a subclass relationship between these terms. Of note, the edge between term A and term B is only valid during the intersection of the stage ranges for these terms. For example, the heart atrium, present from Theiler stage 15 through 28 (or adult), is a part of the heart, which is present for more development stages, Theiler stage 11 through 28. This representation of anatomical structures allows for precise annotation of recombinase activity to facilitate searches. GXD offers an anatomy browser (http://www.informatics.jax.org/vocab/gxd/anatomy) to help identify optimal anatomical terms for searching. As well, the CrePortal search field contains an autofill feature to assist in identifying the desired structure term. The EMAPA-directed acyclic graph structure improves the likelihood of search success by returning all results that are subclasses of the search term. A toggle on the CrePortal search allows users to search by anatomical structures in which the recombinase activity has been detected or assayed, allowing users, if they so choose, to review the structures in which the absence of recombinase activity has been demonstrated. The “and nowhere else” check box allows users to narrow their search to reporters whose activity has been detected in the specified structure (or its substructures) but nowhere else.

Alternatively, the recombinase alleles and transgenes can be searched for based on the driver used to produce recombinase expression. These drivers include endogenous mouse promoters, exogenous once from other species, viral or bacterial promoters, and synthetic ones. Like the recombinase activity search field, the recombinase driven by search field offers an autofill function that lists the matching drivers. The two search fields can be used in conjunction to further refine the selection of recombinase alleles and transgenes returned.

**Fig. 4:**
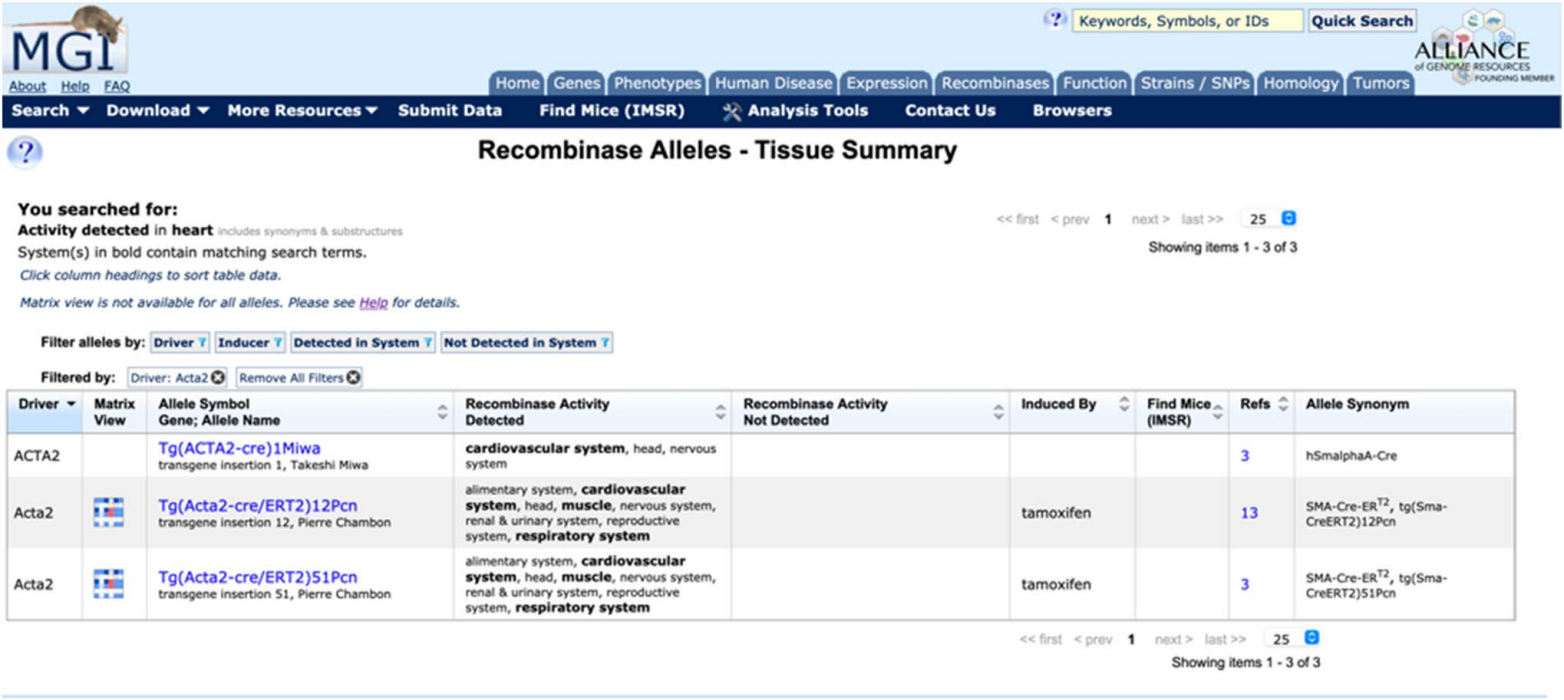
Recombinase Allele-Tissue summary results for a search using the anatomical structure ‘heart.’ Results fields include the driver, allele symbol, recombinase activity structures detected in, recombinase activity structures not detected in, inducing agent (if applicable), link to the International Mouse Strain Resource (IMSR), link to associated references, and allele synonyms

Results of queries by anatomical structure and/or driver produce a summary table of the curator annotated data within the CrePortal for a given recombinase allele or transgene (Fig. 4). Columns contain the driver, allele symbol with link to the allele detail page, recombinase activity structures detected in, recombinase activity structures not detected in, inducing agent (if applicable), link to the International mouse Strain Resource (IMSR), link to associated references, and allele synonyms. Filter functions allow users to limit the results by specific driver, inducer, system detected in, or system not detected in. The result columns also have alpha-numeric sort function.

**Fig. 5:**
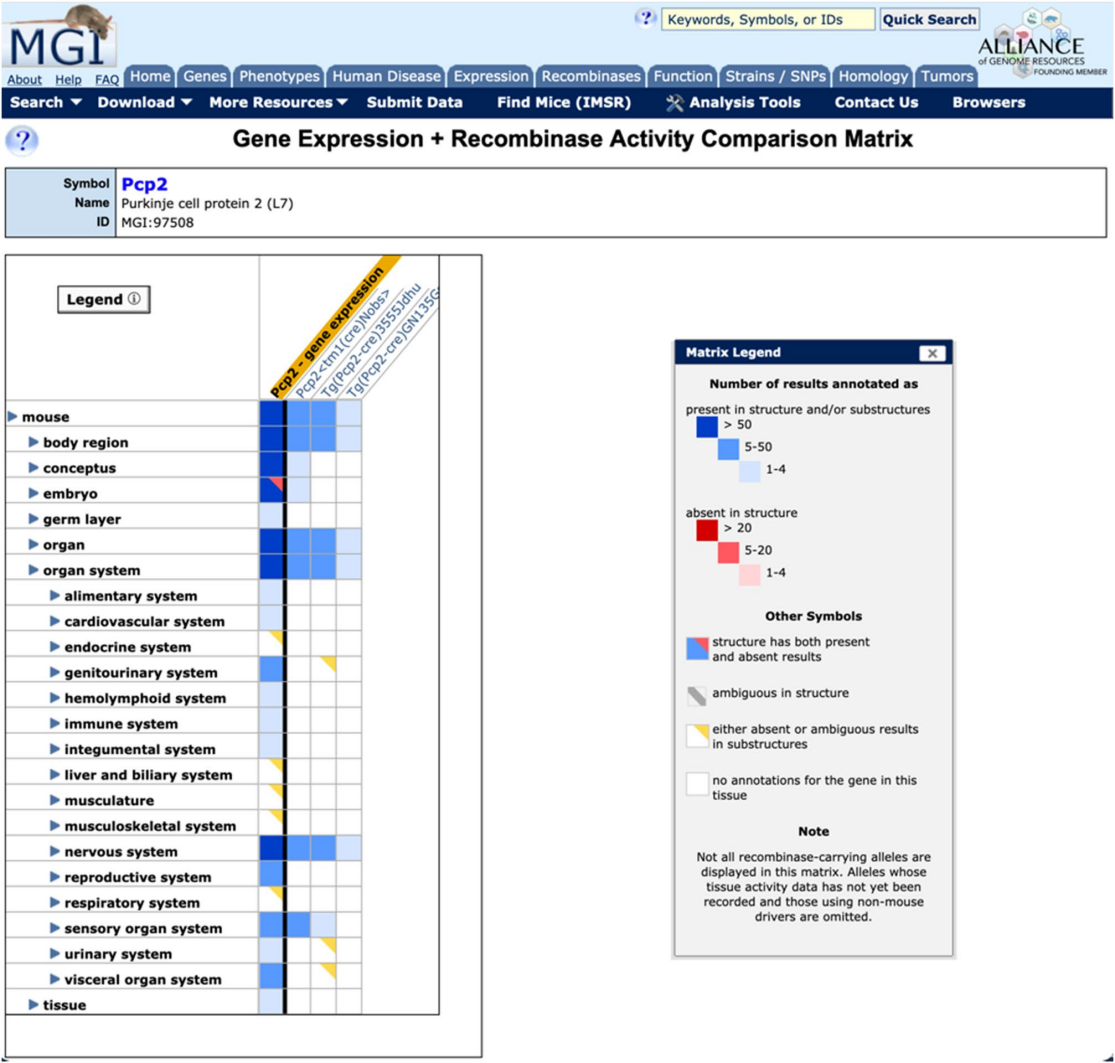
Matrix view of Cre activity from Pcp2 driver lines compared to endogenous gene expression. Shown are the activity assay data for three recombinase-expressing lines utilizing the mouse Pcp2 promoter compared to endogenous Pcp2 gene expression. The EMAPA mouse anatomy ontology is shown on the left and the toggles can be opened and closed to view more substructures for a given system. The red square indicates that activity was specifically assayed in a system but not detected

In addition, a new matrix view that juxtaposes the endogenous expression pattern of a gene (provided by GXD) against activity patterns for recombinases driven by that gene is now available (Fig. 5). This allows users to anticipate recombinase activities in tissues not yet reported for a particular driver of expression, and check whether activity or expression of a particular line is as expected compared to endogenous gene expression.

## Recombinase allele and recombinase activity details

**Fig. 6:**
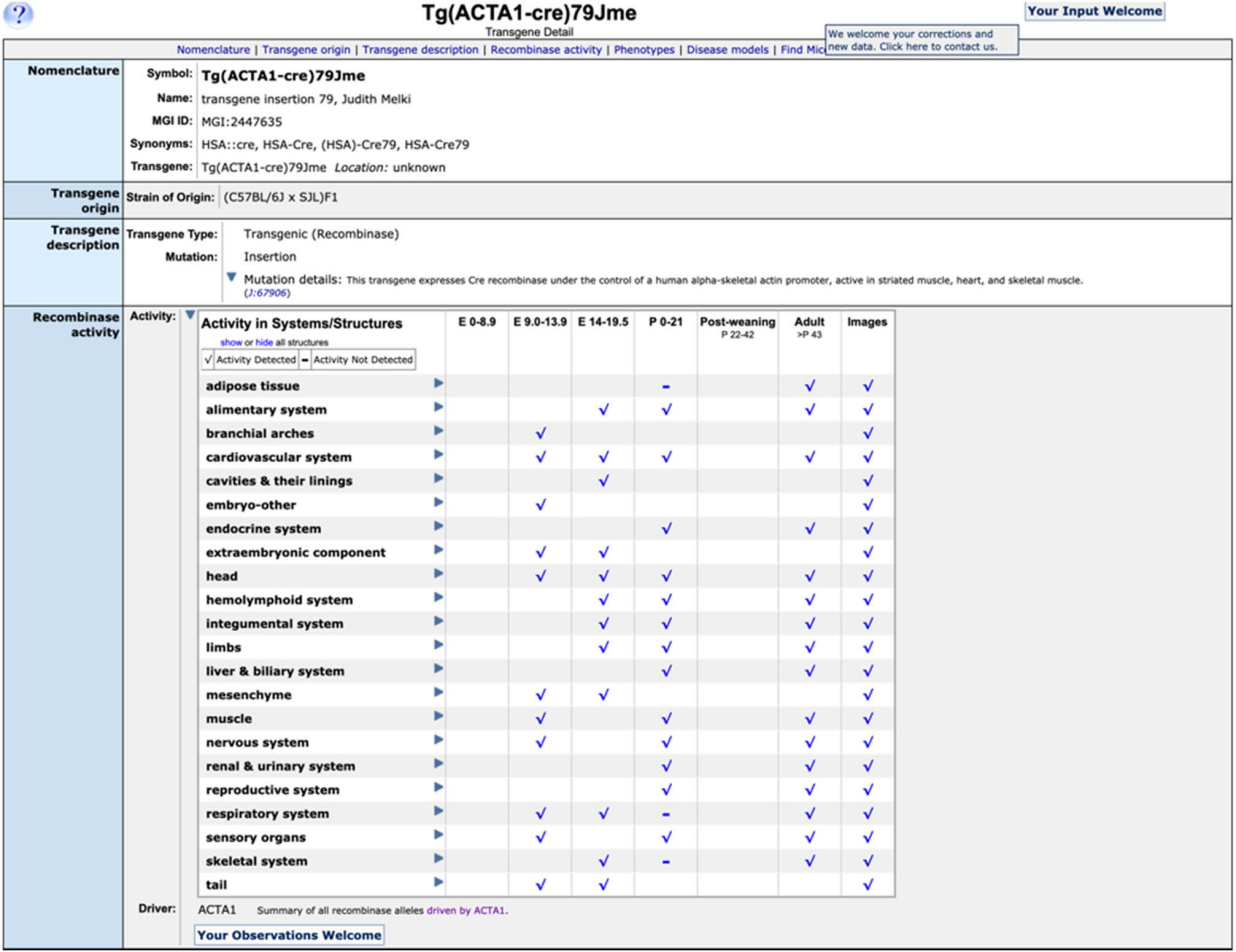
Transgene detail page with opened recombinase activity table and driver information. Expandable systems and structures activity table subdivided by stage ranges. Check marks indicate activity detected; minus signs indicate confirmed absence of activity. When available, links to images for the data are provided. A clickable button to report additional results or issues using this recombinase line is provided at the bottom of the recombinase activity section

Whether accessed through CrePortal search results or MGI’s quick search field, specific, detailed information about individual recombinase alleles and transgenes is presented in conjunction with an expandable grid of systems and structures versus time points derived from annotated recombinase activity (Fig. 6). The recombinase activity grid is shown open when the page is accessed via a CrePortal search results page, whereas it is closed by default, but can be expanded via toggle, when the page is accessed via other search forms available at MGI. Check marks indicate activity detected; minus signs indicate confirmed absence of activity.

**Fig. 7:**
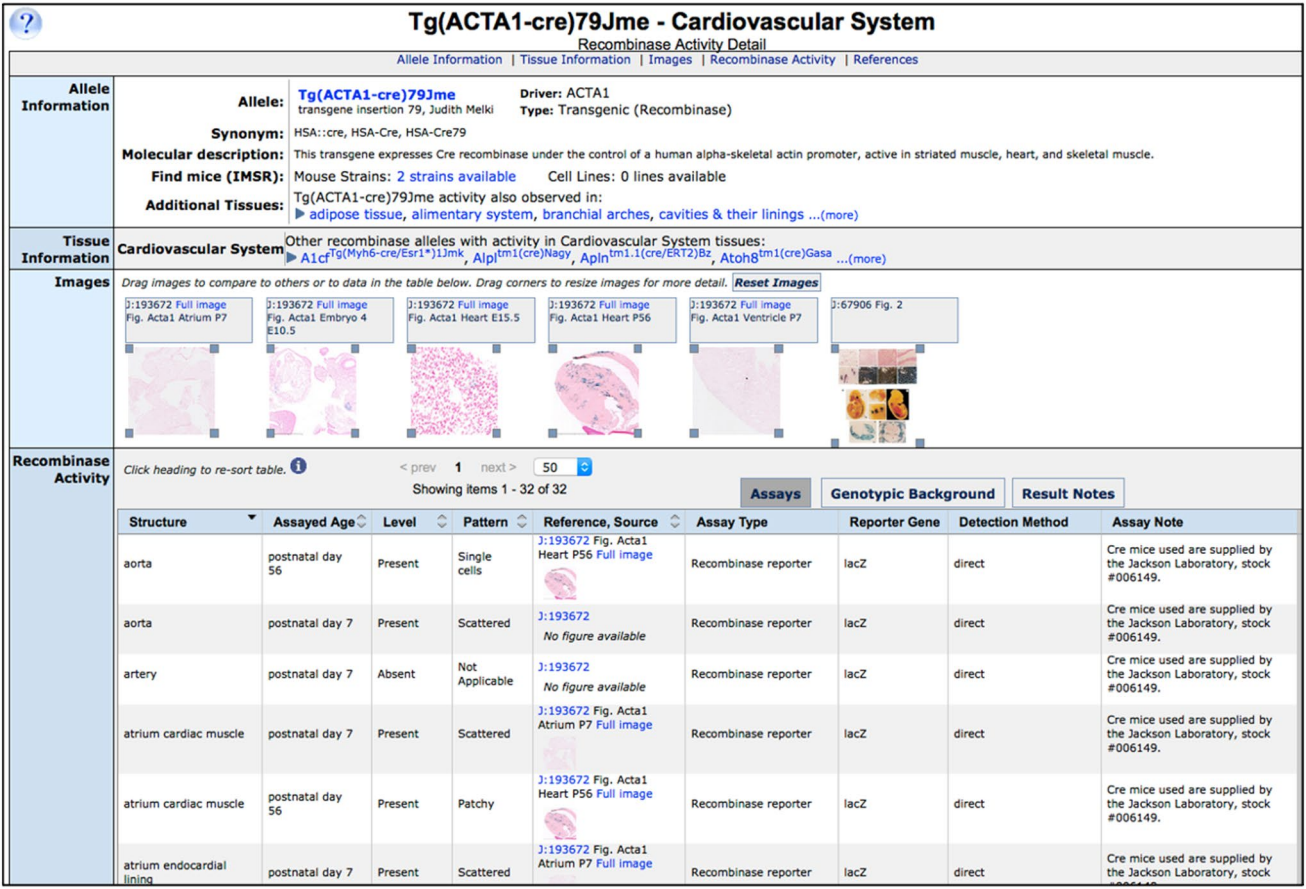
Recombinase activity details with expandable and draggable images. Shown is a cardiovascular system activity detail page for mice expressing Cre under the control of a human ACTA1 driver. In addition to molecular details and availability of mice, other alleles with activity in the cardiovascular system are available as a list. Images are shown and can be expanded by dragging a gray corner of the image and moved to other regions of the page. Activity details include the structure, age, level, and pattern of the detected activity. Assay methods, genotype background, and individual results notes related to the annotation are available by clicking on the tab buttons

Tissue specificity information can be accessed by choosing an anatomical system in the expression summary grid (Fig. 7). The recombinase detail pages have sortable data columns indicating tissue structure, assayed age of specimen, pattern, level, reference, images if available, and assay details with specimen assay notes. Users can resize each image and drag it anywhere on the page. This is especially useful for side-by-side comparisons of expression in each structure.

## Programmatic data access and data submission

The CrePortal provides free public web access to data from http://www.informatics.jax.org/home/recombinase as described above. CrePortal data access is also available through MouseMine (http://www.mousemine.org) (Motenko et al. 2015), an instance of InterMine that offers flexible querying, templates, and iterative querying of results that are integrated with other mouse data provided by MGD and GXD. MouseMine also links to other model organism InterMine instances. MouseMine access is also available via a RESTful API, with client libraries in Perl, Python, Ruby, Java, and JavaScript. Data are also available in tab-delimited format from the CrePortal home page and the MGI reports page (http://www.informatics.jax.org/downloads/reports/index.html#cre).

Finally, the CrePortal supports direct submission from individual investigators and from projects of all images and annotations, ensuring seamless access to all data alongside curated results from the literature. A “Your Observations Welcome” button is available at the bottom of the recombinase activity section of allele detail pages and can be used to report additional activity or problems using the line (Fig. 6). Submissions of novel activity and image data can be made via our submissions form (http://www.informatics.jax.org/mgihome/submissions/recombinase_submission.cgi) or by bulk submission initially via MGI user support. Workflows via programmatic submissions are handled on a case-by-case basis and include the submission of Cre characterization data from the JAX Cre Repository.

## Systematic characterization of recombinase-expressing mouse lines

A significant part of the data in the CrePortal comes from projects that perform a systematic characterization of recombinase-expressing mouse lines, including our work as part of the JAX Cre Repository. Despite the power and versatility of cre driver strains, full awareness and consideration of the caveats and limitations to their use are critical for good experimental design (Schmidt-Supprian and Rajewsky 2007). We (Heffner et al. 2012) and others (Eckardt 2004; Lan et al. 2007; Kucherlapati et al 2006) have shown that Cre lines can exhibit “off-target” activity, that is excision activity in domains not intended or expected based on the predicted function of the driver construct. Inconsistent and/or mosaic deletion activity has been observed (Lu et al. 2008; Means et al. 2005; Pilon et al. 2008) and inducible Cre driver lines, typically estrogen receptor ligand binding domain fusions, can present similar challenges (Feil et al. 1997; Indra et al. 1999; Metzger and Chambon 2001) including leakiness (Balordi and Fishell 2007). In addition, we have reported a phenomenon of widespread, but inconsistent, activation in Cre lines that otherwise display specific activity (Heffner et al. 2012). The engineering approach used to generate a given Cre driver can impact function. For example, transgenics can suffer from integration site-mediated alteration of expression levels and patterns, copy number instability, insertional mutagenesis, and epigenetic silencing of the transgene (Matthaei 2007). We and others have recently shown insertional mutagenesis is common, and this is accompanied by structural rearrangements and/or co-integration of unexpected/unreported passenger sequences (Goodwin et al. 2019; Cain-Hom et al. 2017), which can affect Cre function or the expression of genes at or near the integration site (Laboulaye et al. 2018). Targeting Cre to a specific locus and thus assuring full endogenous regulatory context can improve the fidelity of expression, but haploinsufficiency at a particular locus could confound interpretation of phenotypes if the driver gene is related to the gene or phenotype being investigated (Eagleson et al. 2007). Cre expression in the germline, most commonly the female germline, can impact the degree of excision activity or trigger widespread activation (Gallardo et al. 2007; Lomeli et al. 2000). Together, these observations illustrate the need for systematic evaluation and reporting of Cre driver function and caveats to assure the reliability and reproducibility of studies using these powerful tools.

**Fig. 8:**
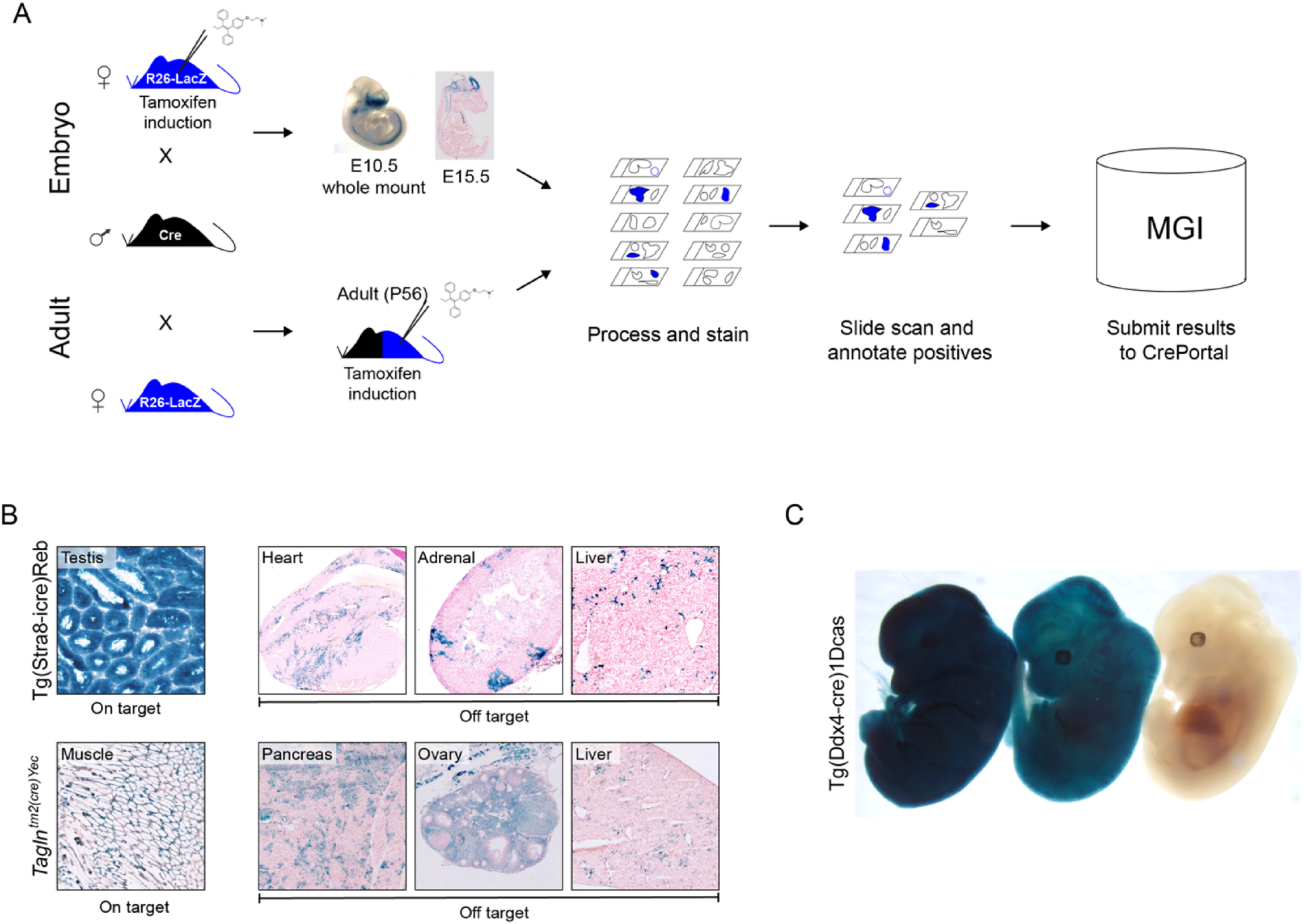
JAX cre driver characterization pipeline. **A** Summary of pipeline illustrating the use of a LacZ reporter and characterization of three timepoints: adult, E15.5, and E10.5. For inducible lines, tamoxifen is used to activate Cre activity in adult cre/reporter F1 animals, or in pregnant reporter females for embryonic time points. Whole mount staining is performed on E10.5 embryos, which are then serially sectioned. For E15.5 and adult time points, fresh frozen sections are stained and slides with positive staining are scanned and annotated. All data are submitted to the CrePortal. **B** Illustration of results from adult samples showing both on-target and off-target activity for commonly used Cre lines. **C** Illustration of variable activity for three embryos from the same Cre line

For highly used lines, the extensive literature provides a reasonably complete picture of the on-target (intended) activity, but frequently this does not include a full assessment of activity in non-target tissues. Existing Cre lines are often used for entirely new research purposes, and depending on the experiment, off-target activity might confound interpretation of results. Therefore, the widespread adoption of individual Cre lines as generalized tools further necessitates systematic evaluation of all potential recombinase actively. Because JAX is the single largest distributor of Cre driver strains, with several hundred unique lines available, we have built an efficient and high-throughput workflow to systematically assess Cre driver functionality using a sensitive reporter strain and characterizing activity in a broad range of tissues at multiple time points (Heffner et al. 2012). Summarized in Fig. 8, the pipeline has been refined to focus on two developmental time points (embryonic days 10.5 and 15.5) and a single adult time point (8 weeks/56 days). For adults, both males and females are included to capture activity in sex-specific organs, including the gonads. Whole E10.5 embryos are LacZ stained, while fresh frozen cryosections are processed and stained for E15.5 embryos and adult tissues. To balance throughput with the need for a broad assessment, we have selected a standard set of 55, 90, and 65 structures for E10.5, E15.5, and adult tissues, respectively. Our use of colorimetric staining allows for a streamlined workflow that includes full slide scanning, which are annotated to anatomical terms where positive staining is observed. Systematic characterization has revealed unexpected/off-target recombination in many Cre alleles in common use (example in 7B), which may confound their use for specific research questions. Moreover, the pipeline has uncovered additional caveats and challenges that could influence the interpretation of results, including variable widespread activation in some individuals (Fig. 8C) (Heffner et al. 2012). Ultimately, our unbiased approach allows individual investigators to determine whether these off-target and/or unexpected effects would influence their approach, including modification of their experimental design (e.g., different driver, additional controls) to account for the limitations of a given strain.

## Discussion

The development of conditional-ready alleles for spatio-temporal or inducible gene knock-out or transgene expression has expanded our knowledge of gene function in the context of both anatomical structure and developmental stage. The ever-increasing availability of recombinase-expressing alleles and transgenes are limited in their usefulness by the inability of researchers to select the optimal recombinase allele or transgene based on their activity patterns and potential phenotypic impact absent complete characterization of the recombination activity. The CrePortal provides an invaluable resource to identify and evaluate the potential location and time of recombination for suitability in a given experiment depending on the researcher’s hypothesis. The availability of both summarized and detailed recombinase activity data with comparison to driver gene expression patterns gives researchers a complete and searchable context for annotated and expected recombinase activity. Furthermore, the CrePortal supports direct submission of data reporting recombinase activity, simplifying centralized access to large-scale Cre driver generation and/or characterization programs.

Future directions for the CrePortal include the retrieval of data associated with specific cell types and improved search and visualization tools. For example, the current anatomical tissue search will not support a search for “Purkinje cell,” an output cell of the cerebellar cortex. The Grid2 and Pcp2 gene drivers express recombinase in Purkinje cells but are annotated to “Purkinje cell layer” in the CrePortal; however, additional cell types exist in this tissue. Furthermore, the striatonigral and striatopallidal projection neurons in the striatum of the brain selectively express the Drd1 and Drd2 dopamine receptors, respectively (Gong et al. 2007). However, mouse lines expressing recombinases under the control of these drivers are both annotated to the tissue “corpus striatum,” obfuscating the cell type specificity of these drivers within this tissue. We will use the Cell Ontology (Diehl et al. 2016) to annotate cell-specific activity in conjunction with anatomical location.

As the number of Cre lines and data continue to increase, it is imperative that there are effective visualization tools to assist with effectively sorting through available information. We will develop additional search and display tools to provide data as aggregate comparisons, with the ability to drill down to specific characterization data. For example, a differential search for a Cre line with expression in tissue X at mid-gestational age and NOT in adult or vice versa may be useful to observe changes in a temporal manner or to study changes in adult tissues when expression in embryo tissues may result in lethality. This will require the addition of developmental stage-specific search capabilities for Cre activity at the portal, similar to the GXD Differential Expression Search interface. We will continue to develop new search and visualization tools as new technologies arise.
